# Poly[[diaqua-μ_6_-succinato-di-μ_5_-succinato-didysprosium(III)] mono­hydrate]

**DOI:** 10.1107/S1600536811024330

**Published:** 2011-06-30

**Authors:** Wei Xu, Hai-Sheng Chang, Xia-Xia Guo

**Affiliations:** a Center of Applied Solid State Chemistry Research, Ningbo University, Ningbo, Zhejiang 315211, People’s Republic of China

## Abstract

The title compound, {[Dy_2_(C_4_H_4_O_4_)_3_(H_2_O)_2_]·H_2_O}_*n*_, is isostructural with other lanthanide succinates of the same formula. The Dy^III^ atom is nine-coordinated in a tricapped trigonal–prismatic environment by eight O atoms, derived from six carboxyl­ate groups and a water mol­ecule. One of the independent succinate anions is located about a crystallographic inversion center and the uncoordinated water mol­ecule lies on a twofold axis. The crystal structure comprises edge-shared DyO_9_ polyhedra linked by succinate bridges, forming a three-dimensional network architecture. Intra- and inter­molecular O—H⋯O hydrogen bonds are present in the crystal structure.

## Related literature

For related compounds, see: Perles *et al.* (2004 ▶); Serpaggi & Ferey (1999 ▶); He *et al.* (2007 ▶); Seguatni *et al.* (2004 ▶); Zhou *et al.* (2005 ▶); Cui *et al.* (2005 ▶); Yu *et al.* (2006 ▶); Li (2007 ▶). 
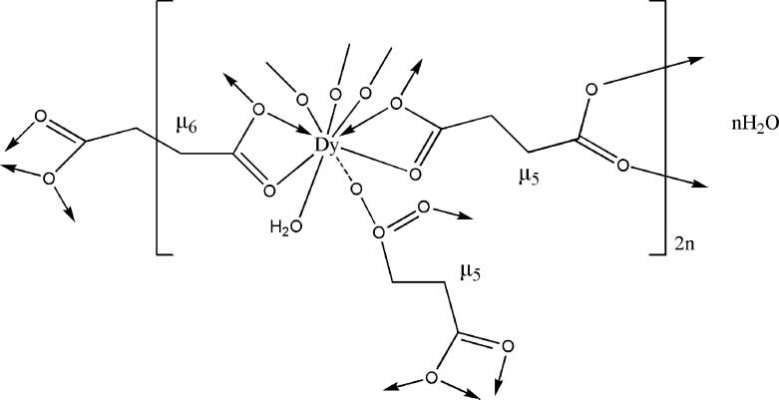


## Experimental

### 

#### Crystal data

[Dy_2_(C_4_H_4_O_4_)_3_(H_2_O)_2_]·H_2_O
*M* *_r_* = 727.26
Monoclinic, 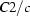

*a* = 19.981 (4) Å
*b* = 7.7616 (16) Å
*c* = 13.868 (3) Å
β = 121.49 (3)°
*V* = 1834.0 (9) Å^3^
*Z* = 4
Mo *K*α radiation
μ = 8.17 mm^−1^
*T* = 293 K
0.45 × 0.20 × 0.14 mm

#### Data collection

Rigaku R-AXIS RAPID diffractometer
Absorption correction: multi-scan (*ABSCOR*; Higashi, 1995 ▶) *T*_min_ = 0.154, *T*_max_ = 0.319
8691 measured reflections
2093 independent reflections
2016 reflections with *I* > 2σ(*I*)
*R*_int_ = 0.018

#### Refinement

*R*[*F* ^2^ > 2σ(*F* ^2^)] = 0.016
*wR*(*F* ^2^) = 0.038
*S* = 1.09
2093 reflections
133 parameters
H-atom parameters constrained
Δρ_max_ = 0.78 e Å^−3^
Δρ_min_ = −0.73 e Å^−3^

### 

Data collection: *RAPID-AUTO* (Rigaku, 1998 ▶); cell refinement: *RAPID-AUTO*; data reduction: *CrystalStructure* (Rigaku/MSC, 2004 ▶); program(s) used to solve structure: *SHELXS97* (Sheldrick, 2008 ▶); program(s) used to refine structure: *SHELXL97* (Sheldrick, 2008 ▶); molecular graphics: *SHELXTL* (Sheldrick, 2008 ▶); software used to prepare material for publication: *SHELXTL*.

## Supplementary Material

Crystal structure: contains datablock(s) global, I. DOI: 10.1107/S1600536811024330/vm2100sup1.cif

Structure factors: contains datablock(s) I. DOI: 10.1107/S1600536811024330/vm2100Isup2.hkl

Additional supplementary materials:  crystallographic information; 3D view; checkCIF report

## Figures and Tables

**Table 1:** Hydrogen-bond geometry (Å, °)

*D*—H⋯*A*	*D*—H	H⋯*A*	*D*⋯*A*	*D*—H⋯*A*
O7—H7*A*⋯O3^i^	0.85	2.07	2.878 (3)	158
O7—H7*B*⋯O2^ii^	0.85	1.86	2.710 (3)	174
O8—H8*W*⋯O7	0.85	2.17	2.949 (4)	153

Symmetry codes: (i) 
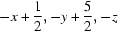
; (ii) 
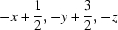
.

**Fig. 1.**
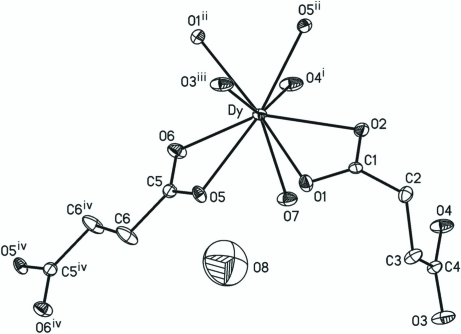
The coordination environment of the DyIII ion in (I), showing the atom labeling and dispacement ellipsoids drawn at the 45% probability level. H atoms have been removed for clarity. [Symmetry codes: (i) -x + 2, -y + 1, -z + 2; (ii) -x, -y, -z + 1; (iii) -x + 1/2, y - 1/2, -z + 1/2; (iv) -x + 1, -y + 2, -z + 1.]

**Fig. 2.**
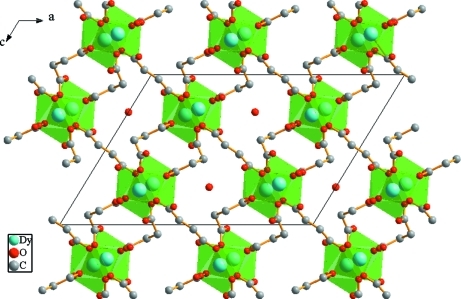
View of the packing in (I), drawing the tricapped trigonal-prismatic geometry of DyIII in green. H atoms have been removed for clarity.

## supplementary crystallographic information

### Comment

There is considerable interest in the study of coordination frameworks with suitable rigid multidentate ligands. Especially those having long chain dicarboxylates present interesting behavior owing to their conformational flexibility and coordination diversity. Lanthanide ions exhibit high affinity for oxygen and diverse coordination modes. In an attempt to further understand the formation of lanthanide–organic framework materials, we present here the hydrothermal synthesis and crystal structure of a new *Ln*–succinate complex, (I).

The title compound, (I), is isostructural with the known *Ln*–succinate complexes where *Ln* = Y and La (Perles *et al.*, 2004), Pr (Serpaggi & Ferey, 1999), Nd (He *et al.*, 2007), Sm (Seguatni *et al.*, 2004), Gd (Zhou *et al.*, 2005), Tb (Cui *et al.*, 2005), Ho (Yu *et al.*, 2006), and Er (Li, 2007) analogs, but it represents the first reported succinate coordination polymer of dysprosium (III).

The asymmetric unit in (I) comprises a Dy atom, one and a half succinate anions, a coordinated water molecule and half an uncoordinated water molecule (Fig. 1). The complete second succinate dianion, containing O5 and O6, is generated from the half-ion by inversion and the uncoordinated water molecule O atom is located on a twofold axis. The Dy^III^ ion is nine-coordinated within a tricapped trigonal-prismatic geometry defined by eight O atoms, derived from six carboxylate anions, and a water molecule. The crystal structure comprises edge–sharing DyO_8_(OH_2_) polyhedra forming chains along the *b*–axis direction by sharing one edge with each neigboring polyhedron. The Dy···Dy distance within chains is 4.046 (1) Å. These chains are in turn linked *via* succinate bridges, forming a three-dimensional framework (Fig. 2.). Intra- and intermolecular O–H···O hydrogen bonds are present in the crystal structure (Table 1).

### Experimental

A mixture of Dy(NO_3_)_3_.7H_2_O (0.1316 g, 0.30 mmol), succinic acid (0.0354 g, 0.30 mmol), 1,10-phenanthroline (0.0595 g, 0.30 mmol), water (10 ml) was adjusted to a pH = 5.25 by NaOH solution. The mixture was sealed in a Teflon-lined stainless steel reactor and heated at 443 K for 3 d. After the reaction system had cooled slowly to room temperature, a small quantity of colourless crystals was isolated.

### Refinement

H atoms bonded to C atoms were placed in their geometrically calculated positions and refined using the riding model, with C–H distances 0.97 Å and *U*_iso_(H) = 1.2 *U*_eq_(C). H atoms attached to O atoms were found in a difference Fourier map and then refined using the riding model, with O–H distances fixed as initially found and with O–H distances 0.85 Å and *U*_iso_(H) values set at 1.2 *U*_eq_(O).

### Crystal data

**Table:**

[Dy_2_(C_4_H_4_O_4_)_3_(H_2_O)_2_]·H_2_O	*F*(000) = 1368
*M**_r_* = 727.26	*D*_x_ = 2.634 Mg m^−^^3^
Monoclinic, *C*2/*c*	Mo *K*α radiation, λ = 0.71073 Å
Hall symbol: -C 2yc	Cell parameters from 8350 reflections
*a* = 19.981 (4) Å	θ = 3.0–27.5°
*b* = 7.7616 (16) Å	µ = 8.17 mm^−^^1^
*c* = 13.868 (3) Å	*T* = 293 K
β = 121.49 (3)°	Prism, colorless
*V* = 1834.0 (9) Å^3^	0.45 × 0.20 × 0.14 mm
*Z* = 4	

### Data collection

**Table:**

Rigaku R-AXIS RAPID diffractometer	2093 independent reflections
Radiation source: fine-focus sealed tube	2016 reflections with *I* > 2σ(*I*)
graphite	*R*_int_ = 0.018
ω scans	θ_max_ = 27.5°, θ_min_ = 3.0°
Absorption correction: multi-scan (*ABSCOR*; Higashi, 1995)	*h* = −25→25
*T*_min_ = 0.154, *T*_max_ = 0.319	*k* = −10→9
8691 measured reflections	*l* = −17→17

### Refinement

**Table:**

Refinement on *F*^2^	Secondary atom site location: difference Fourier map
Least-squares matrix: full	Hydrogen site location: inferred from neighbouring sites
*R*[*F*^2^ > 2σ(*F*^2^)] = 0.016	H-atom parameters constrained
*wR*(*F*^2^) = 0.038	*w* = 1/[σ^2^(*F*_o_^2^) + (0.0112*P*)^2^ + 12.5672*P*] where *P* = (*F*_o_^2^ + 2*F*_c_^2^)/3
*S* = 1.09	(Δ/σ)_max_ = 0.005
2093 reflections	Δρ_max_ = 0.78 e Å^−^^3^
133 parameters	Δρ_min_ = −0.73 e Å^−^^3^
0 restraints	Extinction correction: *SHELXL97* (Sheldrick, 2008), Fc^*^=kFc[1+0.001xFc^2^λ^3^/sin(2θ)]^-1/4^
Primary atom site location: structure-invariant direct methods	Extinction coefficient: 0.00050 (4)

### Special details

**Table:**

Geometry. All e.s.d.'s (except the e.s.d. in the dihedral angle between two l.s. planes) are estimated using the full covariance matrix. The cell e.s.d.'s are taken into account individually in the estimation of e.s.d.'s in distances, angles and torsion angles; correlations between e.s.d.'s in cell parameters are only used when they are defined by crystal symmetry. An approximate (isotropic) treatment of cell e.s.d.'s is used for estimating e.s.d.'s involving l.s. planes.
Refinement. Refinement of *F*^2^ against ALL reflections. The weighted *R*-factor *wR* and goodness of fit *S* are based on *F*^2^, conventional *R*-factors *R* are based on *F*, with *F* set to zero for negative *F*^2^. The threshold expression of *F*^2^ > σ(*F*^2^) is used only for calculating *R*-factors(gt) *etc*. and is not relevant to the choice of reflections for refinement. *R*-factors based on *F*^2^ are statistically about twice as large as those based on *F*, and *R*- factors based on ALL data will be even larger.

### Fractional atomic coordinates and isotropic or equivalent isotropic displacement parameters (Å^2^)

**Table:**

	*x*	*y*	*z*	*U*_iso_*/*U*_eq_	
Dy	0.268767 (7)	0.716662 (17)	0.229964 (11)	0.01104 (6)	
O1	0.18695 (12)	0.9822 (3)	0.13564 (17)	0.0161 (4)	
O2	0.16984 (14)	0.7651 (3)	0.02657 (18)	0.0179 (4)	
O3	0.19605 (15)	1.2559 (3)	−0.1745 (2)	0.0225 (5)	
O4	0.18010 (14)	0.9767 (3)	−0.1583 (2)	0.0214 (5)	
C1	0.15245 (16)	0.9157 (4)	0.0371 (2)	0.0118 (5)	
C2	0.09015 (17)	1.0166 (4)	−0.0632 (2)	0.0152 (6)	
H2A	0.0506	1.0544	−0.0473	0.018*	
H2B	0.0648	0.9413	−0.1286	0.018*	
C3	0.12259 (19)	1.1741 (4)	−0.0920 (3)	0.0167 (6)	
H3A	0.0791	1.2478	−0.1429	0.020*	
H3B	0.1556	1.2388	−0.0231	0.020*	
C4	0.16978 (17)	1.1315 (4)	−0.1461 (2)	0.0131 (5)	
O5	0.32431 (11)	0.9822 (3)	0.34063 (17)	0.0142 (4)	
O6	0.40702 (13)	0.7716 (3)	0.3808 (2)	0.0195 (5)	
C5	0.39496 (16)	0.9239 (4)	0.3932 (2)	0.0131 (5)	
C6	0.46055 (17)	1.0439 (4)	0.4690 (3)	0.0240 (7)	
H6A	0.4498	1.0936	0.5237	0.029*	
H6B	0.4624	1.1373	0.4239	0.029*	
O7	0.33328 (14)	0.8880 (3)	0.15135 (19)	0.0200 (5)	
H7A	0.3304	0.9973	0.1475	0.024*	
H7B	0.3319	0.8471	0.0935	0.024*	
O8	0.5000	0.9782 (13)	0.2500	0.123 (3)	
H8W	0.4592	0.9198	0.2324	0.147*	

### Atomic displacement parameters (Å^2^)

**Table:**

	*U*^11^	*U*^22^	*U*^33^	*U*^12^	*U*^13^	*U*^23^
Dy	0.01269 (8)	0.00799 (8)	0.01316 (8)	−0.00046 (5)	0.00725 (6)	−0.00019 (5)
O1	0.0165 (10)	0.0156 (10)	0.0126 (9)	0.0015 (8)	0.0050 (8)	−0.0025 (8)
O2	0.0279 (12)	0.0099 (10)	0.0135 (10)	0.0003 (9)	0.0091 (9)	0.0001 (8)
O3	0.0366 (14)	0.0109 (10)	0.0342 (13)	0.0015 (9)	0.0285 (12)	0.0030 (9)
O4	0.0332 (13)	0.0104 (10)	0.0346 (13)	0.0020 (9)	0.0274 (11)	0.0002 (9)
C1	0.0120 (13)	0.0136 (14)	0.0121 (12)	−0.0052 (11)	0.0079 (11)	−0.0001 (11)
C2	0.0122 (13)	0.0200 (15)	0.0132 (13)	0.0005 (11)	0.0064 (11)	0.0030 (12)
C3	0.0235 (15)	0.0137 (14)	0.0195 (14)	0.0058 (12)	0.0157 (13)	0.0048 (12)
C4	0.0159 (13)	0.0105 (13)	0.0144 (13)	0.0015 (11)	0.0088 (11)	0.0026 (11)
O5	0.0083 (9)	0.0153 (10)	0.0146 (9)	0.0004 (8)	0.0030 (8)	−0.0041 (8)
O6	0.0127 (10)	0.0118 (10)	0.0261 (12)	0.0008 (8)	0.0046 (9)	−0.0003 (9)
C5	0.0103 (13)	0.0148 (14)	0.0130 (12)	−0.0017 (11)	0.0054 (11)	0.0012 (11)
C6	0.0108 (14)	0.0159 (15)	0.0325 (18)	−0.0014 (12)	0.0024 (13)	−0.0079 (14)
O7	0.0320 (13)	0.0134 (11)	0.0247 (11)	−0.0033 (9)	0.0219 (10)	−0.0024 (9)
O8	0.090 (6)	0.136 (8)	0.139 (7)	0.000	0.058 (6)	0.000

### Geometric parameters (Å, °)

**Table:**

Dy—O4^i^	2.312 (2)	C2—C3	1.531 (4)
Dy—O5^ii^	2.414 (2)	C2—H2A	0.9700
Dy—O1^ii^	2.417 (2)	C2—H2B	0.9700
Dy—O3^iii^	2.434 (2)	C3—C4	1.517 (4)
Dy—O5	2.461 (2)	C3—H3A	0.9700
Dy—O7	2.467 (2)	C3—H3B	0.9700
Dy—O6	2.480 (2)	O5—C5	1.286 (3)
Dy—O2	2.486 (2)	O5—Dy^iv^	2.414 (2)
Dy—O1	2.529 (2)	O6—C5	1.236 (4)
O1—C1	1.275 (3)	C5—C6	1.500 (4)
O1—Dy^iv^	2.417 (2)	C6—C6^vi^	1.508 (6)
O2—C1	1.249 (4)	C6—H6A	0.9700
O3—C4	1.257 (4)	C6—H6B	0.9700
O3—Dy^v^	2.434 (2)	O7—H7A	0.8500
O4—C4	1.246 (4)	O7—H7B	0.8501
O4—Dy^i^	2.312 (2)	O8—H8W	0.8503
C1—C2	1.513 (4)		
			
O4^i^—Dy—O5^ii^	75.88 (8)	O6—Dy—C1	133.45 (8)
O4^i^—Dy—O1^ii^	77.11 (8)	O2—Dy—C1	25.28 (8)
O5^ii^—Dy—O1^ii^	68.86 (8)	O1—Dy—C1	25.96 (7)
O4^i^—Dy—O3^iii^	144.52 (8)	C5—Dy—C1	112.44 (9)
O5^ii^—Dy—O3^iii^	74.58 (8)	C1—O1—Dy^iv^	155.0 (2)
O1^ii^—Dy—O3^iii^	74.22 (8)	C1—O1—Dy	93.77 (18)
O4^i^—Dy—O5	130.94 (8)	Dy^iv^—O1—Dy	109.71 (8)
O5^ii^—Dy—O5	152.29 (2)	C1—O2—Dy	96.55 (17)
O1^ii^—Dy—O5	106.58 (7)	C4—O3—Dy^v^	134.7 (2)
O3^iii^—Dy—O5	77.89 (7)	C4—O4—Dy^i^	145.7 (2)
O4^i^—Dy—O7	73.12 (8)	O2—C1—O1	118.4 (3)
O5^ii^—Dy—O7	134.18 (7)	O2—C1—C2	121.5 (3)
O1^ii^—Dy—O7	132.93 (8)	O1—C1—C2	120.0 (3)
O3^iii^—Dy—O7	142.35 (7)	O2—C1—Dy	58.17 (15)
O5—Dy—O7	69.79 (7)	O1—C1—Dy	60.27 (15)
O4^i^—Dy—O6	85.79 (8)	C2—C1—Dy	178.43 (19)
O5^ii^—Dy—O6	138.96 (7)	C1—C2—C3	113.3 (2)
O1^ii^—Dy—O6	71.41 (8)	C1—C2—H2A	108.9
O3^iii^—Dy—O6	104.14 (9)	C3—C2—H2A	108.9
O5—Dy—O6	52.36 (7)	C1—C2—H2B	108.9
O7—Dy—O6	70.81 (8)	C3—C2—H2B	108.9
O4^i^—Dy—O2	83.01 (8)	H2A—C2—H2B	107.7
O5^ii^—Dy—O2	70.51 (7)	C4—C3—C2	114.3 (3)
O1^ii^—Dy—O2	137.94 (7)	C4—C3—H3A	108.7
O3^iii^—Dy—O2	104.93 (9)	C2—C3—H3A	108.7
O5—Dy—O2	114.41 (7)	C4—C3—H3B	108.7
O7—Dy—O2	72.92 (8)	C2—C3—H3B	108.7
O6—Dy—O2	143.72 (8)	H3A—C3—H3B	107.6
O4^i^—Dy—O1	128.17 (8)	O4—C4—O3	124.9 (3)
O5^ii^—Dy—O1	104.58 (7)	O4—C4—C3	117.9 (3)
O1^ii^—Dy—O1	152.80 (2)	O3—C4—C3	117.2 (3)
O3^iii^—Dy—O1	78.58 (8)	C5—O5—Dy^iv^	151.66 (19)
O5—Dy—O1	66.36 (7)	C5—O5—Dy	93.61 (17)
O7—Dy—O1	71.21 (7)	Dy^iv^—O5—Dy	112.19 (8)
O6—Dy—O1	115.49 (7)	C5—O6—Dy	93.98 (17)
O2—Dy—O1	51.24 (7)	O6—C5—O5	119.6 (3)
O4^i^—Dy—C5	107.57 (9)	O6—C5—C6	121.9 (3)
O5^ii^—Dy—C5	157.37 (7)	O5—C5—C6	118.5 (3)
O1^ii^—Dy—C5	89.81 (8)	O6—C5—Dy	60.34 (15)
O3^iii^—Dy—C5	92.88 (8)	O5—C5—Dy	59.60 (15)
O5—Dy—C5	26.79 (8)	C6—C5—Dy	174.0 (2)
O7—Dy—C5	66.22 (8)	C5—C6—C6^vi^	112.9 (3)
O6—Dy—C5	25.67 (8)	C5—C6—H6A	109.0
O2—Dy—C5	131.74 (8)	C6^vi^—C6—H6A	109.0
O1—Dy—C5	90.89 (8)	C5—C6—H6B	109.0
O4^i^—Dy—C1	105.65 (8)	C6^vi^—C6—H6B	109.0
O5^ii^—Dy—C1	87.20 (8)	H6A—C6—H6B	107.8
O1^ii^—Dy—C1	154.72 (7)	Dy—O7—H7A	122.1
O3^iii^—Dy—C1	92.12 (8)	Dy—O7—H7B	116.7
O5—Dy—C1	90.70 (8)	H7A—O7—H7B	110.3
O7—Dy—C1	70.00 (8)		

Symmetry codes: (i) −*x*+1/2, −*y*+3/2, −*z*; (ii) −*x*+1/2, *y*−1/2, −*z*+1/2; (iii) *x*, −*y*+2, *z*+1/2; (iv) −*x*+1/2, *y*+1/2, −*z*+1/2; (v) *x*, −*y*+2, *z*−1/2; (vi) −*x*+1, −*y*+2, −*z*+1.

### Hydrogen-bond geometry (Å, °)

**Table:**

*D*—H···*A*	*D*—H	H···*A*	*D*···*A*	*D*—H···*A*
O7—H7A···O3^vii^	0.85	2.07	2.878 (3)	158
O7—H7B···O2^i^	0.85	1.86	2.710 (3)	174
O8—H8W···O7	0.85	2.17	2.949 (4)	153

Symmetry codes: (vii) −*x*+1/2, −*y*+5/2, −*z*; (i) −*x*+1/2, −*y*+3/2, −*z*.

## References

[bb1] Cui, G. H., Li, J. R. & Bu, X. H. (2005). *J. Mol. Struct.* **740**, 187–191.

[bb2] He, Y.-K., Wang, X.-F., Zhang, L.-T., Han, Z.-B. & Ng, S. W. (2007). *Acta Cryst.* E**63**, m3019.

[bb3] Higashi, T. (1995). *ABSCOR* Rigaku Corporation, Tokyo, Japan.

[bb4] Li, F. (2007). *Acta Cryst.* E**63**, m840–m841.

[bb5] Perles, J., Iglesias, M., Ruiz-Valero, C. & Snejko, N. (2004). *J. Mater. Chem.* **14**, 2683–2698.

[bb6] Rigaku (1998). *RAPID-AUTO* Rigaku Corporation, Tokyo, Japan.

[bb7] Rigaku/MSC (2004). *CrystalStructure* Rigaku/MSC Inc., The Woodlands, Texas, USA.

[bb8] Seguatni, A., Fakhfakh, M., Vauley, M. J. & Jouini, N. (2004). *J. Solid State Chem.* **177**, 3402–3410.

[bb9] Serpaggi, G. M. & Ferey, G. (1999). *Micropor. Mesopor. Mat.* **32**, 311–318.

[bb10] Sheldrick, G. M. (2008). *Acta Cryst.* A**64**, 112–122. 10.1107/S0108767307043930 18156677

[bb11] Yu, B., Wang, X.-Q., Wang, R.-J., Shen, G.-Q. & Shen, D.-Z. (2006). *Acta Cryst.* E**62**, m1620–m1622.

[bb12] Zhou, Y. F., Jiang, F. L., Yuan, D. Q., Wu, B. L. & Hong, M. C. (2005). *J. Mol. Struct.* **743**, 21–27.

